# The effects of virtual reality on pain relief in ICU patients: meta-analysis and systematic review

**DOI:** 10.3389/fmed.2026.1792073

**Published:** 2026-05-19

**Authors:** Ping Zhao, Huineng Xiao, Deping Lü, Min Feng, Xihui Sun

**Affiliations:** 1 Hemodialysis Department, Hemodialysis Department, Bishan Hospital of Chongqing Medical University, Chongqing, China; 2 Affiliated Hospital of North Sichuan Medical College, Nanchong, China; 3 Nanchong Central Hospital Affiliated to North Sichuan Medical College, Nanchong, China; 4 Nursing Department, Bishan Hospital of Chongqing Medical University (Bishan Hospital of Chongqing), Chongqing, China; 5 Department of Nursing Science, Faculty of Nursing, Universiti Malaya, Kuala Lumpur, Malaysia

**Keywords:** intensive care unit, meta-analysis, non-pharmacological analgesia, pain management, virtual reality

## Abstract

**Background:**

Pain is common in conscious ICU patients, with over 50% experiencing mild to severe pain from procedures, disease, or routine care. Virtual reality (VR) interventions may help reduce pain, but evidence remains inconsistent, and their effect on analgesic use is unclear. The purpose of this systematic review and meta-analysis is to evaluate the impact of virtual reality technology or intervention on pain levels and analgesic dosage in adult ICU patients.

**Methods:**

Relevant literature published from January 2015 to February 2026 in PubMed and eight other electronic databases has been comprehensively searched. The Cochrane Risk of Bias Tool and Joanna Briggs Institute checklist were applied for quality assessment of randomized controlled trials (RCTs) and quasi-experimental studies (QES), respectively. The data analysis was conducted using R studio (4.5.2). The GRADE system was employed to evaluate the certainty of evidence.

**Results:**

Thirteen studies involving 979 patients were included. Meta-analysis of seven clinical studies (7 RCTs) demonstrated that head-mounted VR devices delivering 360°relaxing nature scenes significantly reduced pain scores compared to conventional care[*I^2^* = 56.5%, *SMD* = −0.66, 95% CI (−0.93, −0.40), *p* = 0.032; low-certainty evidence]. The effect was most pronounced during acute procedural pain, particularly postoperative chest drain removal. Only 5 studies reported post-enrolment analgesic and sedative doses, and heterogeneity of reporting precluded pooled estimates of opioid- or sedative-sparing effects.

**Conclusion:**

Nature-based, distraction-focused VR appears to confer additional analgesic benefit for ICU patients, especially around brief high-intensity procedures. However, given the complexity of pain sources in the ICU, variations in study design, and the diversity of VR content, its true impact on ICU pain and the demand for analgesic medications should still be continually validated.

**Systematic review registration:**

https://www.crd.york.ac.uk/PROSPERO/view/CRD420251231605, PROSPERO, registration number: CRD420251231605.

## Introduction

Intensive care unit (ICU) patients often experience severe pain, particularly following invasive treatments and surgeries (1). Studies show that over 50% of ICU patients suffer from mild to severe pain due to surgeries, routine care, or underlying conditions (2, 3). These pains not only affect their physical condition, but also exacerbate their psychological distress (4, 5). Specifically, procedural pain such as chest drain removal, endotracheal intubation, or traumatic wound dressing changes are major sources of suffering, often reaching pain scores of 7–9 on the Numeric Rating Scale (NRS) (6, 7). The impact of pain in ICU patients extends far beyond subjective experience. Uncontrolled pain is closely linked to numerous adverse outcomes, including higher infection rates, longer mechanical ventilation durations, delirium, extended hospitalization, and increased mortality (8). Additionally, unresolved pain may lead to chronic pain, functional impairment, and post-traumatic stress disorder (PTSD), further diminishing quality of life and affecting social reintegration (9). Furthermore, pain-induced functional avoidance syndrome in critically ill patients manifests as diminished engagement in early mobilization, pulmonary rehabilitation, and therapeutic interventions due to nociceptive hypersensitivity, establishing a self-perpetuating nociception-avoidance-deconditioning triad that significantly compromises ICU recovery trajectories (10). Therefore, effective pain management is crucial not only for immediate comfort but also for long-term health and recovery.

Although current pain management strategies primarily depend on pharmacological treatments, particularly opioids, their side effects such as respiratory depression, delirium, tolerance, and dependency present significant risks for ICU patients (11). This has led to a critical need for non-pharmacological pain relief methods, particularly in the high-demand ICU environment (12). Multimodal pain management strategies, combining pharmacological and non-pharmacological interventions, are widely recommended to reduce drug use and enhance pain relief (13).

Virtual reality (VR) has emerged as an innovative non-pharmacological pain relief method, showing great potential in alleviating pain, reducing anxiety, and improving treatment tolerance (14). By immersing patients in a virtual environment, VR effectively diverts attention, diminishing pain perception (15). Several studies have demonstrated significant efficacy of VR in relieving acute pain, particularly in oncology, trauma, and post-surgical patients (16–18). However, despite some research on VR applications for ICU patients, current evidence remains heterogeneous regarding the implementation context, VR intervention content, and effectiveness evaluation (19–23).

The application of VR in ICU patients, particularly in complex clinical scenarios like post-surgical recovery, still faces many uncertainties and challenges (24). While some studies suggest that VR effectively reduces pain scores, its durability, impact on opioid use, and applicability to different clinical settings have yet to be fully validated (25, 26). Additionally, VR’s efficacy may be influenced by factors such as the patient’s cognitive status, baseline pain management protocols, timing of intervention, and the type of virtual content used (27).

Crucially, there remains a conspicuous absence of dedicated systematic reviews and meta-analyses examining the impact of VR technology on both pain alleviation and analgesic consumption in ICU settings (28). Therefore, a comprehensive systematic review and meta-analysis of the effectiveness of VR interventions in ICU pain relief is essential.

This study aims to systematically evaluate and perform a meta-analysis of the application of VR technology in ICU pain management, focusing on its impact on pain intensity, analgesic requirements, and related anxiety. The study will also explore potential factors influencing VR’s effectiveness, such as intervention frequency, clinical context, and device type, and assess its combined effect with traditional pharmacological pain relief methods. By providing integrated quantitative evidence on VR’s role in pain management, this research will support the development of more personalized, low-drug-dependency, and patient-centred pain management strategies for critical care.

## Methods

### Reporting and registration protocol

The aim is to systematically review and evaluate the impact of VR intervention on pain relief and analgesic use in ICU patients through systematic literature retrieval and meta-analysis. This systematic review was registered with PROSPERO (CRD420251231605) and conducted following the Preferred Reporting Items for Systematic Reviews and Meta-Analyses (PRISMA) guidelines (29).

### Databases and search strategy

Two researchers (DP and HN) independently performed conducted a comprehensive search across multiple databases, including PubMed, Scopus, the Cochrane Library, Web of Science, China National Knowledge Infrastructure, the Cumulative Index to Nursing and Allied Health Literature, Wanfang, Wiley Online Library, and ScienceDirect, to identify relevant randomized controlled trials (RCTs), quasi-experimental studies (QES), and feasibility studies examining the application of VR in ICU patients. This study only included relevant literature published between January 2015 and February 2026. A comprehensive search was conducted using English terms including: “virtual reality,” “VR therapy,” “immersive virtual reality”, and “virtual reality intervention” (intervention terms); “intensive care unit”, “ICU patients”, “critically ill”, and “critical illness” (population terms); “pain relief,” “pain management,” “analgesia,” “opioid-sparing,” and “pain intensity” (outcome terms); as well as “randomised controlled trial”, “RCT”, “clinical trial”, and “quasi-experimental study” (study design terms). The search strategy was specifically tailored to identify studies evaluating VR’s efficacy for pain management in ICU settings, excluding rehabilitation-focused interventions to maintain relevance to the research question. For details about search strategy, please refer to Supplementary File 1. In case of any disputes regarding the final inclusion of literature, a third evidence-based researcher will be consulted to reach a final agreement.

### Inclusion criteria

The items for inclusion eligibility were as follows: (1) studies published in any language; (2) adult ICU patients; (3) randomized controlled trials (RCTs), quasi-experimental studies (QES) or feasibility studies; (4) any form of VR intervention (e.g., VR meditation, VR music, 360° relaxing videos or VR games) compared with standard ICU care (e.g., usual nursing care or routine pharmacological analgesia); and (5) outcomes related to pain intensity and/or post-enrolment analgesic and sedative drug use.

### Exclusion criteria

Exclusion criteria were: (i) pediatric critically ill or non-routine hospitalized patients; (ii) non-original studies; (iii) inability to obtain the full text; (iv) irrelevant outcome reports; and (v) VR protocol bias.

### Study selection criteria

We conducted an initial search, removed duplicate records using EndNote X9, and screened titles and abstracts to assess relevance. Records were then categorized as “include,” “exclude,” or “uncertain.” For uncertain articles, full texts were retrieved to determine eligibility. Two authors independently evaluated the identified literature. Any disagreements were resolved through consultation with a third author for a final decision.

### Data extraction

Two independent reviewers (ZP and HN) jointly developed the data extraction form and reached consensus on the extraction process and quality assessment. Extracted data included: first author, year, country, patient age/type, study design, sample size, intervention, comparator, reporting of analgesic and sedative use, and pain assessment tools. The primary meta-analytic outcomes were pain scores during the ICU stay and analgesic consumption.

### Quality appraisal

The risk of bias in each RCT and QES article was assessed by two researchers (DP and HN) using Cochrane recommended risk assessment criteria (30). The evaluation criteria include seven elements including random method and hidden allocation. For the QES literature, the Joanna Briggs Institute (JBI) tool (31) was used for assessment. This tool comprises nine items (e.g., baseline comparability, presence of a control group and methods of outcome assessment), each scored as one point. Further details on the risk-of-bias instruments used are provided in Supplementary File 2. To enhance consistency and accuracy, two investigators with expertise in evidence-based medicine independently performed data extraction and quality appraisal, after which inter-rater reliability was calculated to assess agreement (32). Additionally, a third expert in evidence-based medicine will resolve any significant discrepancies that may arise.

### Clinical evidence grading

Using the GRADE framework (GRADE profiler 3.6.1), two researchers evaluated evidence certainty across outcomes, with RCTs initially classified as high-quality and observational studies as low-quality evidence. The assessment considered five key domains: (1) risk of bias, (2) inconsistency, (3) indirectness, (4) imprecision, and (5) publication bias, with final evidence certainty graded as very low, low, moderate, or high for each outcome (33).

### Data synthesis

The meta-analysis was conducted using R studio (4.5.2), assessing continuous variables with standardized mean difference (S*MD*) and 95% confidence interval (*CI*). Heterogeneity was evaluated using the Q test. A random-effects model was used if *I^2^* > 50% and *p* < 0.05; otherwise, a fixed-effects model was applied (34). Sensitivity analysis was performed in R by varying the meta-analytic model, and publication bias was evaluated with funnel plots and Egger’s test.

## Results

### Study screening process and search summary

**Figure 1:**
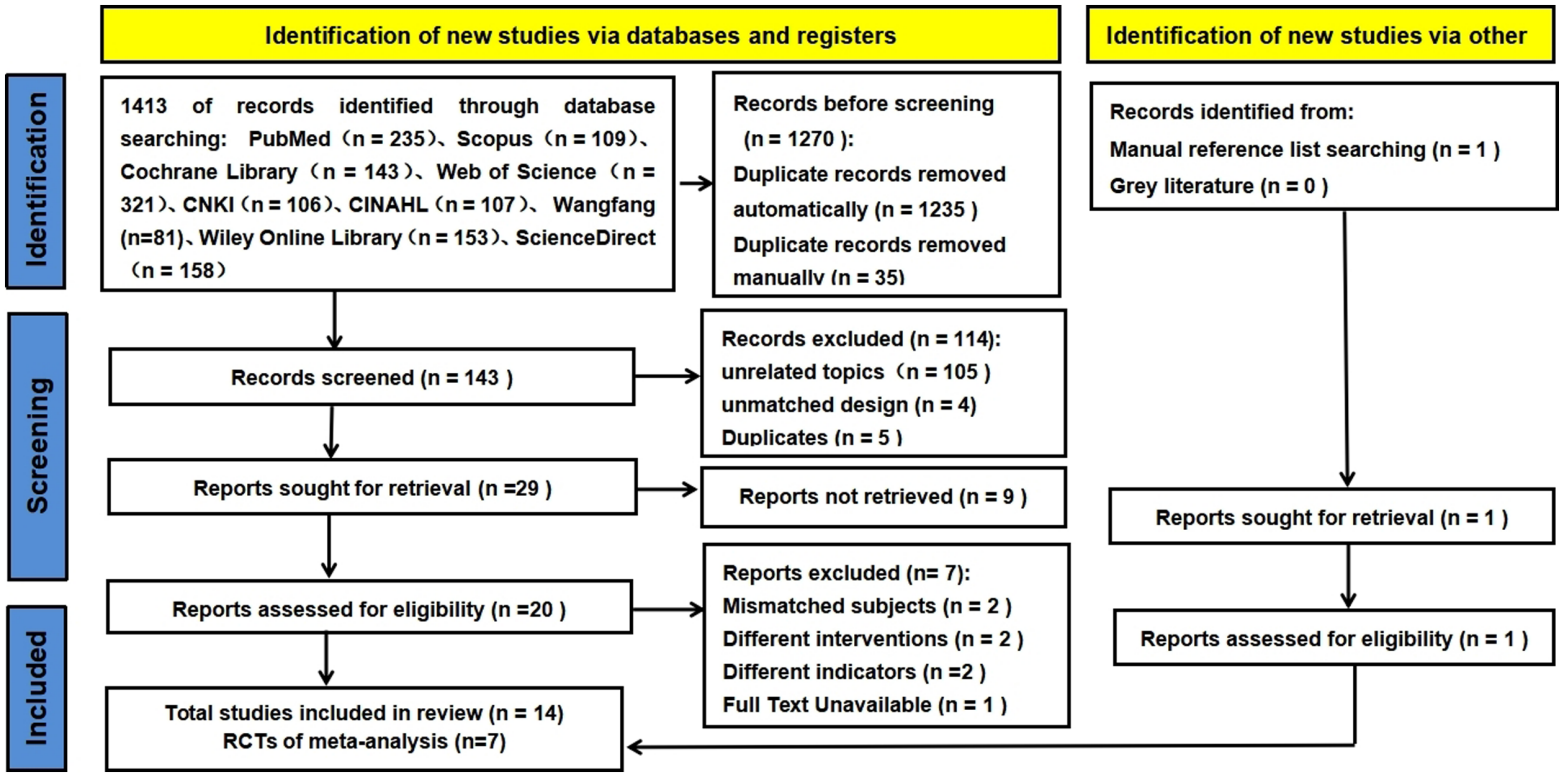
Study screening process (based on PRISMA 2020).

EndNote X9 is used to integrate retrieved citations. First, a total of 1,413 studies were identified through online literature searches. After removing duplicates and applying exclusion criteria, 143 studies remained. 19 studies were selected for full-text review. 14 studies (RCT = 9, QES = 5) were ultimately deemed suitable for inclusion in this meta-analysis and systematic review (19–23, 35–43). The screening of the study is shown in Figure 1.

### Study characteristics

The included studies showed diverse geographical distribution: six were conducted in the USA (*n* = 3) (38, 42, 43) and France (*n* = 3) (23, 40, 41), a further five in China (*n* = 2) (20, 36) and Turkey (*n* = 3) (19, 21, 37), and the remaining three in Belgium, Egypt and Iran (22, 35, 39). Three trials focused on chest drain removal after cardiac surgery, whereas the others applied VR during routine ICU care. Most were undertaken in cardiothoracic, surgical or mixed ICUs, and predominantly enrolled awake, non-intubated patients.

Regarding VR content, most studies used head-mounted devices to deliver relaxation-based interventions, such as viewing real or computer-generated 360° natural scenes, VR-guided meditation or interactive VR games. Intervention frequency ranged from 1 to 7 sessions, although most studies administered a single session lasting 5–15 min. Pain outcomes were typically assessed using the Numeric Pain Rating Scale (NPRS), Visual Analogue Scale (VAS), Numeric Pain Score (NPS) or similar tools. Supplementary qualitative data from mixed-methods studies incorporated patient-reported experience questionnaires. Comprehensive intervention details are presented in Table 1.

**Table 1:** Characteristics of included studies.

Author/Year/Country	Sample E/C A(I)	Study design	Age/Patient type	Intervention program		Sedation or analgesia after admission	Pain measurement tools and results
				Experimental group	Control group		
				Content, frequency, point and duration of VR			
Kızıl, 2026 (19) Türkiye	30:30	RCT	65.3 ± 12.0:64.3 ± 14.9 Stable non-mechanically ventilated patients aged 18 and above.	Immersive VR video: The VR intervention involved 20-min nature-based relaxation scenes viewed through a Google Daydream headset, aimed at calming ICU patients during common invasive procedures. Once only.	Usual care	NR	VAS; VR significantly reduced pain and anxiety in ICU patients compared to standard care, enhancing overall comfort during invasive procedures.
Mohamed Ahmad et al., 2025 (35) Egypt	60:60	RCT	18–60; Alert ICU patients (non-intubated) in ED/general units.	Immersive VR video: Researchers administered a 15-min immersive VR session using Samsung Gear VR (featuring 360°nature scenes), with vital signs, pain, and anxiety levels monitored pre-intervention, immediately post-intervention, and at 30/60-min intervals.	Relax through deep breathing	Post-enrollment analgesic users excluded.	NPS; Smartphone VR session significantly reduced pain scores in ICU patients within 60 min, shifting more from moderate to mild/no pain.
Ju et al., 2025 (36) China	45 (32)	QES	19–83; Perioperative surgical ICU patients.	Immersive VR video: Natural landscape VR with meditation guidance (Huawei smartphone + Cardboard headset) administered for 6 min 15 s to semi-recumbent SICU patients on day 2, with clinical monitoring and assessments at 10 min pre- and post-intervention.	NA	No use of sedative and analgesic drugs was reported.	VAS; 6-min VR nature meditation reduced pain,depression,anxiety and boosted comfort in SICU patients.
Özbaş et al., 2024 (37) Türkiye	20:20	RCT	58–72; Patients undergoing open-heart surgery.	Immersive VR video: During chest tube removal, patients in semi-Fowler’s position viewed a self-selected nature VR video (6 options, 3D visuals/audio) via smartphone-compatible headset. Pain/anxiety were assessed pre-procedure, during, 10-min and 1-h post-procedure.	Usual care	Analgesia given (1 VR, 8 control).	VAS; VR significantly alleviates postoperative pain and reduces analgesic requirements in ICU.
Locke et al., 2024 (38) USA	35 (20)	QES	61 ± 17; Medical intensive care population.	Immersive virtual reality: Meta Quest Pro headset delivering three immersive scenarios (urban/nature/meditation) for passive viewing (5–15 min, mean 10 ± 3 min), single session with researcher monitoring.	NA	NR	VAS; VR reduced pain by 1.3 points (*p* = 0.003), alleviating acute pain in ICU patients.
Dalir et al., 2024 (39) Iran	35:35	RCT	52.3 ± 11.5:54.5 ± 8.3; Patients undergoing.	Immersive virtual reality: VR (Shinecon) with self-selected nature video initiated 5 min pre-CTR until removal completion (~5 min duration). Pain assessed pre-CTR, immediately post-CTR, and 15 min post-CTR.	Usual care	NR	VAS; VR reduced tube-removal pain (effect size 1.1–1.4) with less analgesia
Han et al., 2023 (20) Taiwan, China	35:35	RCT	66 (58.8–77); Postoperative surgical patients.	Immersive VR intervention: within 48 h of admission, patients received the “Grasp Fruit” game twice daily (20 min each, 3 days), sitting in bed to perform fruit-grabbing tasks.	Usual care	NR	CPOT; VR demonstrated no significant improvement in patients’ pain levels overall.
Yesilot et al., 2022 (21) Türkiye	55:55	RCT	19–62; Post-gastrectomy patients.	Immersive relaxation VR training: Patient watch panoramic videos based on oceans, mountains, and forests through VR glasses, and the intervention was performed once on the first day after surgery, (30 ± 5) min.	Usual care and analgesia.	NR	NPRS; VR adjuvant therapy reduced opioid requirements by 23% while improving pain scores in post-gastrectomy critical care patients (*p* = 0.01).
Rousseaux et al., 2022 (22) Belgium	15:22	RCT	66 ± 11.5; Perioperative cardiac surgical patients.	Immersive relaxation VR training: Patients view 360° natural landscapes (lakeside sunrise and relaxation in the clouds) through VR devices, once before and once after surgery, each session lasting 20 min. The intervention lasted for 3 days.	Usual care	Analgesia given (10 in VR, 15 in control)	VAS; VR/hypnosis showed no added benefit for post-cardiac surgery pain or opioid reduction.
Merliot-Gailhoustet et al., 2022 (40) France	53:56	RCT	62(51–69); Alert general ICU patients.	Immersive relaxation VR training: patients watch four videos (Norway, mountainous countryside, India and seaside) through VR glasses for breathing control and hypnosis. Two sessions of VR training, each lasting 15 min (total 60 min).	Regular relaxation methods.	NR	NPRS; VR interventions significantly reduced pain, anxiety, and discomfort in ICU patients
Laghlam et al., 2021 (23) France	90:90	RCT	68(60–78); Post-cardiac surgery patients.	Immersive relaxation VR training: Patients relaxed in a VR-based natural environment (snowy mountains, hot air balloon ride, kayaking) four times: once after randomization, before surgery, 5 min before and 10 min after drain removal (≥15 min per session). The intervention was delivered twice, totaling ≥30 min.	Nitrous oxide/oxygen analgesia	Postoperative IV nefopam 120 mg/day unless contraindicated.	NRS; VR and nitrous oxide both effectively relieved pain with comparable efficacy and safety, though without statistically significant differences in outcomes.
Roxburgh et al., 2021 (41) France	48:51	QES	63 ± 10.9:64.5 ± 10.4; AF ablation patients.	Immersive relaxation VR training: patients achieve breathing training, music therapy and game interaction in 5 computer-simulated VR scenes through VR headset during AFA procedure. The specific frequency and period of intervention are unclear.	Usual care	Morphine; VR: 4.2 ± 1.3 mg; Control: 4.1 ± 1.5 mg	VAS; VR increased subjective comfort without decreasing analgesic use.
Jawed et al., 2021 (42) USA	15(14)	QES	60.8 ± 10.9; Alert general ICU patients.	Immersive VR training: Single 15-min VR beach relaxation session (extendable to 30 min) at 30° semi-recumbency, with immediate post-intervention anxiety/pain assessment under clinician monitoring.	NA	NR	Likert scale; VR elicited mixed reports: half noted no effect, others subjective pain reduction
Ong et al., 2020 (43) USA	59(46)	QES	50 ± 18; Conscious non-intubated SICU patients	Immersive relaxation VR training: Daily VR meditation (5–20 min/session, max 7 sessions) using Daydream, with immediate pain evaluation. 46 participants completed 1–7 sessions (mode = 1–2).	NA	NR; As-needed analgesic medication only	DVPRS; VR reduced opioid use and patient-reported benefits, but lacked statistical significance in objective measures.

### Methodological details

**Figure 2:**
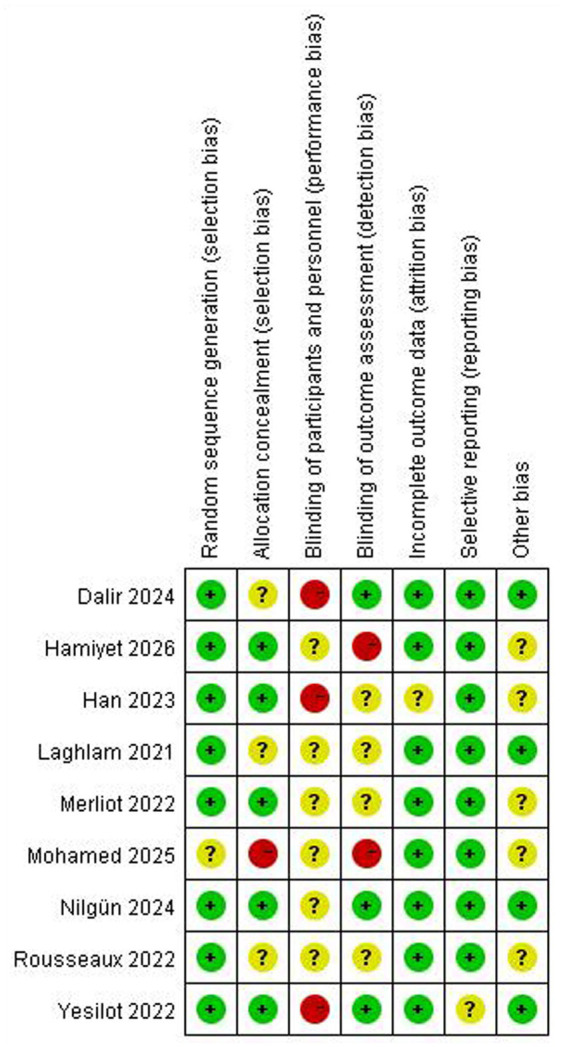
Risk bias of included RCTs.

Two independent investigators (ZP and HN) systematically evaluated all included studies, demonstrating complete concordance in data extraction and quality assessment. Of the 8 randomized controlled trials analysed, 7 documented appropriate randomization procedures while only 4 implemented allocation concealment (20, 21, 37, 40), with merely 3 incorporating blinded outcome assessment—collectively indicating predominant moderate risk of bias (21, 37, 40). Quality appraisal of the 5 quasi-experimental studies using the JBI instrument revealed only 2 studies satisfied the predefined quality threshold (score ≥7/9) (38, 41). The methodological limitations observed across these studies suggest constrained evidentiary robustness. Comprehensive bias evaluation and study characteristics are detailed in Figure 2 and Tables 2, 3 respectively.

**Table 2:** Risk of bias assessment for RCTs.

References	Random method	Allocation concealment	Blind method		Data integrity	Selective reporting	Other bias	Overall bias
			For subjects and intervener	For outcome surveyor				
Kızıl, 2026 (19)	Low	Low	Unclear	High	Low	Low	Unclear	Moderate
Mohamed Ahmad et al., 2025 (35)	Unclear	High	Unclear	High	Low	Low	Unclear	High
Özbaş et al., 2024 (37)	Low	Low	Unclear	Low	Low	Low	Low	Moderate
Dalir et al., 2024 (39)	Low	Unclear	High	Unclear	Low	Low	Low	Moderate
Yesilot et al., 2022 (21)	Low	Low	High	Low	Low	Low	Low	Moderate
Rousseaux et al., 2022 (22)	Low	Unclear	Unclear	Unclear	Low	Low	Unclear	Moderate
Merliot-Gailhoustet et al., 2022 (40)	Low	Low	Unclear	Low	Low	Low	Low	Low
Han et al., 2023 (20)	Low	Low	Unclear	Unclear	Low	Low	Unclear	Moderate
Laghlam et al., 2021 (23)	Low	Unclear	Unclear	Unclear	Low	Low	Low	Moderate

**Table 3:** Quality evaluation of QES studies.

Items/Author	Ju et al., 2025 (36)	Locke et al., 2024 (38)	Roxburgh et al., 2021 (41)	Jawed et al., 2021 (42)	Ong et al., 2020 (43)
Clear articulation of causality	1	1	0	0	1
Comparability of baseline data	0	0	1	0	0
Consistency in standard interventions	1	1	1	1	1
Establishment of a control group	0	0	1	0	0
Diverse measurements before and after intervention	0	1	0	1	1
Completeness of follow-up	1	1	1	1	0
Consistency in outcome assessment methods	1	1	1	1	1
Reliability of outcome assessment methods	1	1	1	1	1
Appropriate data analysis	1	1	1	1	1
Total score	6	7	7	6	6

The tools are derived from the JBI’s assessment items for quasi-experimental study.

### Meta-analysis result of pain score

**Figure 3:**
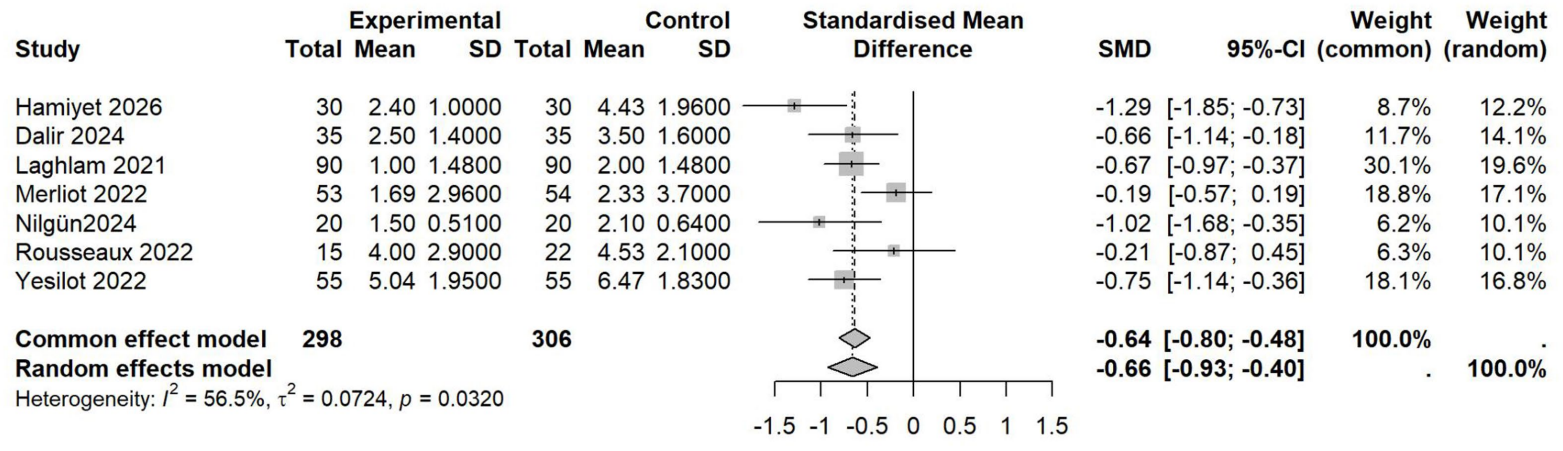
Forest plot of pain score.

Seven studies with 604 patients were included in the synthesis (19, 21–23, 37, 39, 40). The results of the heterogeneity test indicated moderate heterogeneity, with *I^2^* = 56.5%. The random-effects model analysis indicated that VR group showed more significant improvement in pain relief [*SMD* = −0.66, 95% *CI* (−0.93, −0.40), *p* = 0.032; Figure 3]. Due to differences in research protocols and assessment tools, subgroup analysis could not be implemented.

### Heterogeneity and publication bias analysis

**Figure 4:**
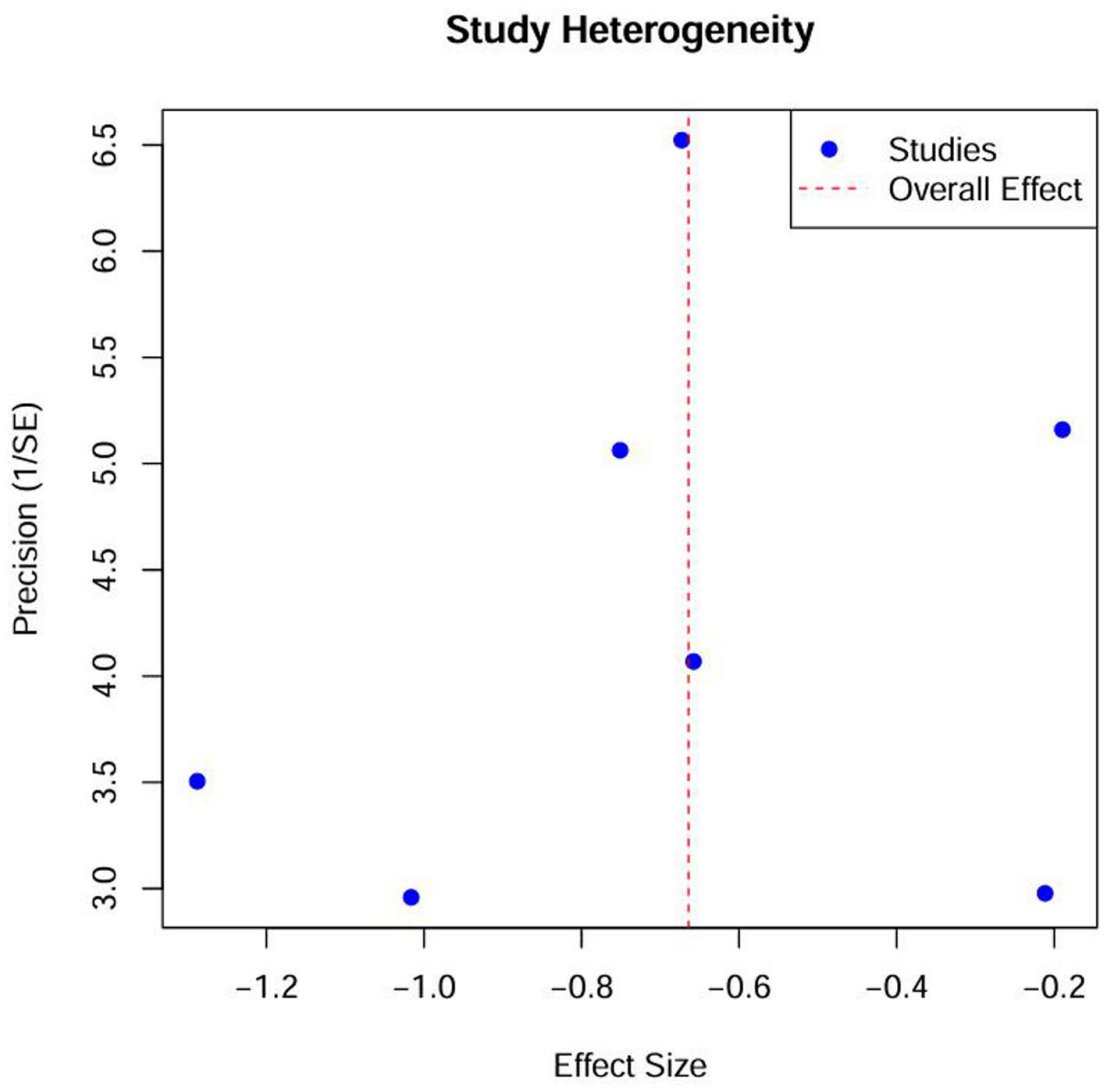
Heterogeneity test diagram.

**Figure 5:**
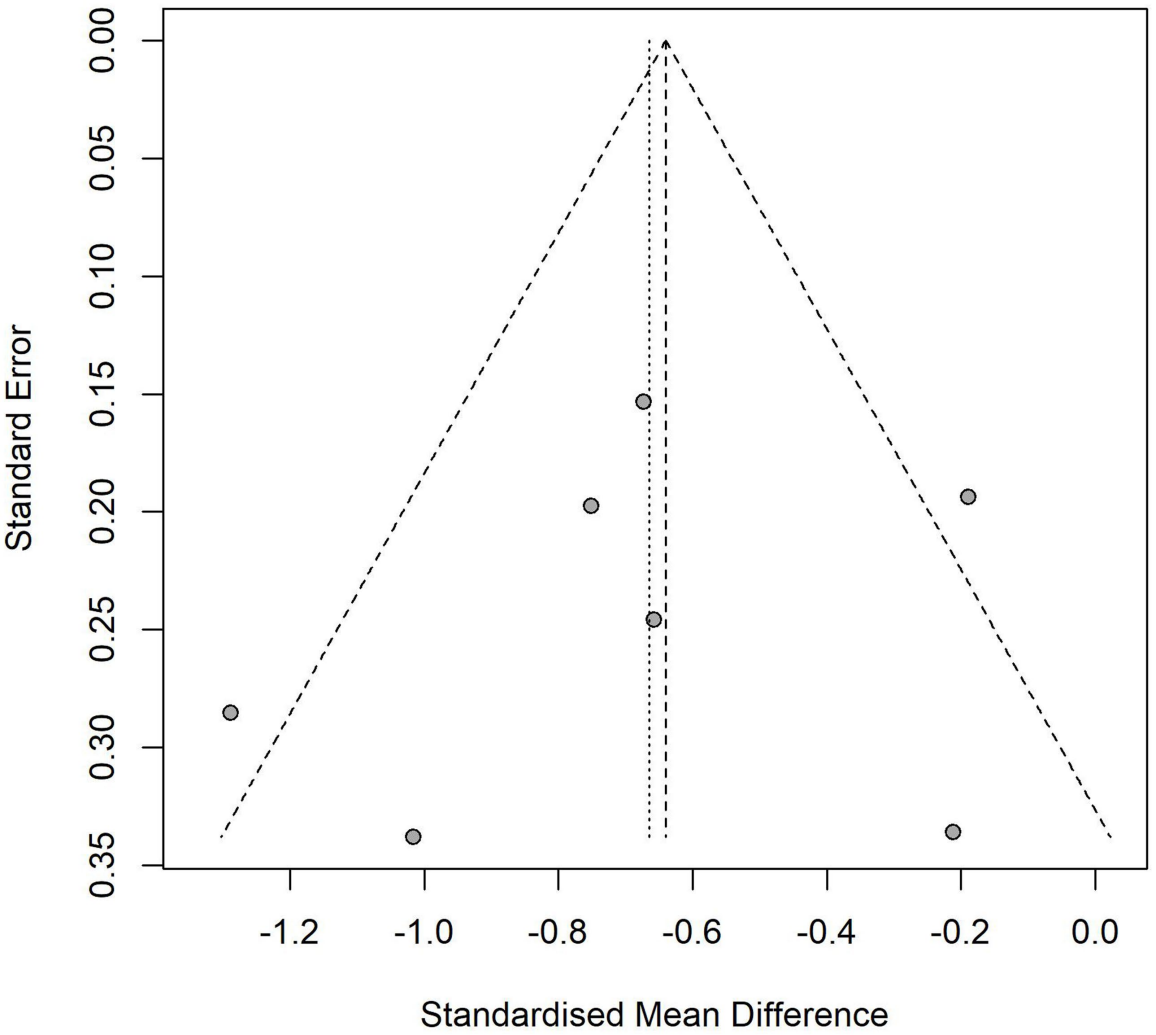
Funnel plot of pain score.

**Figure 6:**
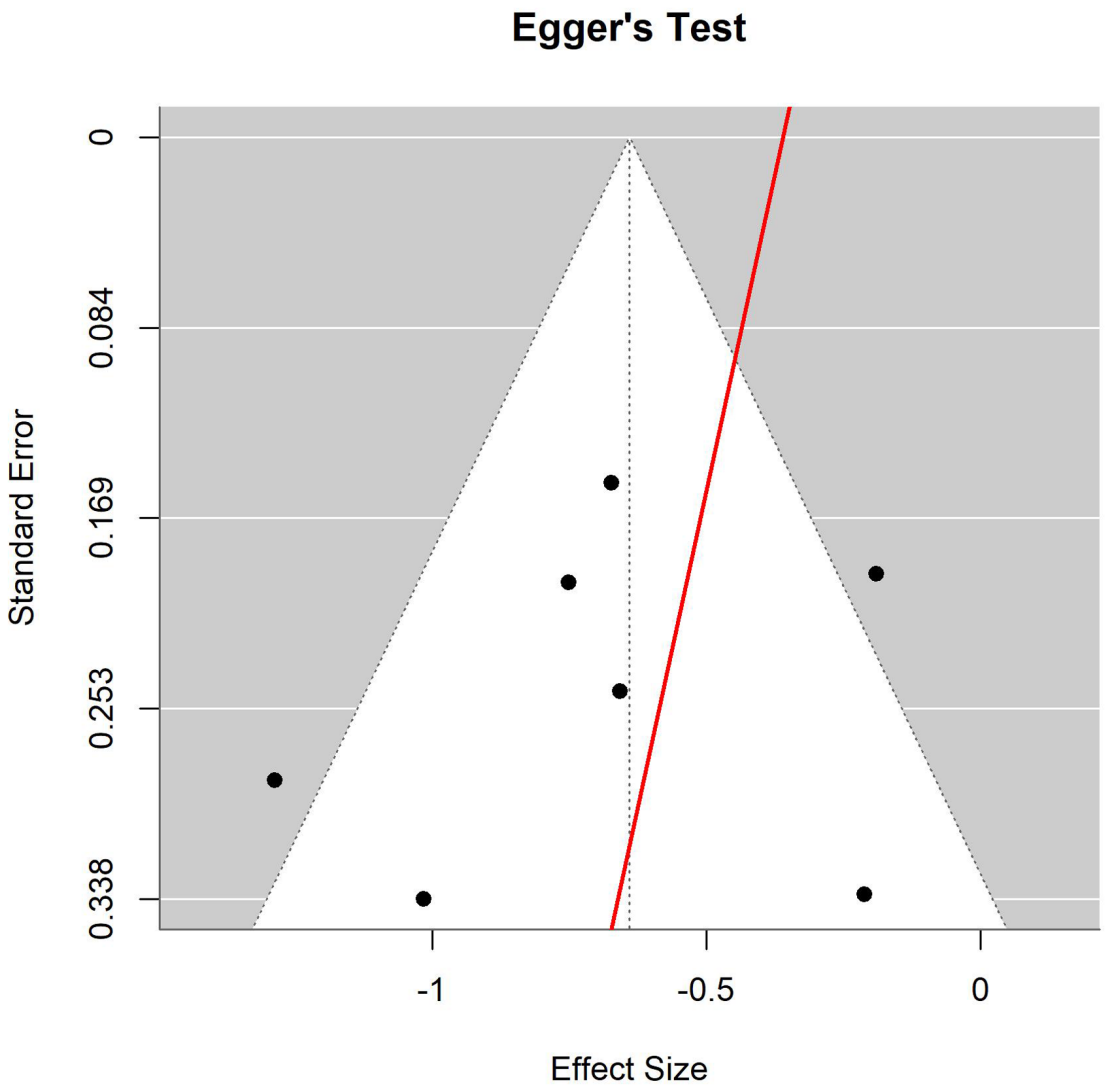
Egger regression test plot of pain score.

The heterogeneity test (Figure 4) showed that the effect of VR intervention varies across studies, with significant differences likely due to factors such as study population, VR content, intervention frequency, and outcome measurement. The funnel plot (Figure 5) revealed that most data points clustered around the center, with no significant rightward skew, suggesting minimal publication bias. However, the Egger regression analysis plot (Figure 6) suggests the presence of some publication bias, indicating that there may be unpublished negative results. Therefore, the findings of this meta-analysis should be interpreted with caution.

### Grading of evidence for meta-analysis results

According to the GRADE rating, the certainty of evidence that VR can improve ICU pain in this meta-analysis is low, suggesting the need for further high-quality research (44). The results of the two reviewers’ independent grading of the evidence were entirely similar.

## Discussion

This systematic review may be the first dedicated meta-analysis examining the efficacy of VR technology for pain management in ICU settings, incorporating 9 RCTs and 5 QES. The primary analysis of 7 pain-focused RCT (19, 21–23, 35, 37, 39, 40) demonstrated statistically significant pain relief (*p* < 0.05) with VR interventions (360° naturalistic videos/immersive experiences) compared to standard care, particularly during high-intensity procedures (e.g., chest tube removal) and acute procedural pain episodes. However, interventions targeting background pain through relaxation/gaming showed inconsistent effects (20), suggesting VR’s preferential utility for procedural analgesia. Methodologically, the majority of RCTs lacked detailed descriptions of blinding procedures (19, 22, 23, 35, 39), while quasi-experimental studies were limited to single-arm designs (36, 38, 42, 43), reflecting inherent methodological limitations in the current evidence base. The GRADE system’s classification of low certainty for this meta-analysis primarily stems from heterogeneity in study designs, participant populations, and VR intervention protocols. Consequently, conclusions regarding VR’s significant efficacy in alleviating ICU pain should be interpreted with due caution.

The current evidence highlights two primary clinical applications of VR for pain management in ICU settings. For acute procedural pain during interventions such as chest tube removal or post-cardiac surgery procedures (23, 37, 39), VR interventions initiated 5–10 min prior to the procedure demonstrate consistent analgesic effects, with RCTs showing significant pain reduction and decreased opioid requirements (39). A second group comprises awake, non-intubated ICU patients experiencing background pain and discomfort during routine care or the recovery phase. In these studies, VR typically consisted of nature scenes, meditation-style videos or low-intensity interactive games, delivered as a single or short series of sessions (22, 35, 40). QES suggest that patients with mild–moderate pain may achieve an approximate 1-point reduction on subjective pain scales, but advantages in objective indices and longer-term outcomes remain uncertain (36, 38, 41). Most trials enrolled awake, non-intubated ICU patients and excluded those who were moderately sedated or mechanically ventilated, likely because VR protocols relied on self-report of pain (19, 35, 37, 39). Additionally, only 5 of the 14 included studies clearly reported post-enrolment analgesic and sedative regimens (22, 23, 36, 37, 41), precluding definitive assessment of potential synergistic effects between pharmacological analgesia and VR interventions in ICU pain management.

Notably, it is well established that ICU patients experience highly heterogeneous pain phenotypes, encompassing acute procedural pain, background pain, mixed pain syndromes, and disease-related chronic pain, each modulated by complex interactions of physiological, psychological, and environmental factors (2, 45). Current evidence remains limited by inadequate characterization of pain aetiologies (35, 36, 40, 43), with most studies applying VR interventions predominantly to postoperative or procedural patients while relying on unidimensional subjective measures (19, 21, 36, 37, 39). These methodological and design limitations may potentially confound the true therapeutic efficacy of VR interventions across diverse ICU pain management scenarios. Given the complexity of pain etiology in the ICU and the high proportion of intubated patients, extrapolating the present findings to more severely ill or ventilated populations is not justified (46). These observations highlight the need for future work, under standardized light sedation–analgesia protocols, to more rigorously establish the efficacy of VR as a comfort-enhancing, non-pharmacological adjunct in ICU pain management. Nevertheless, the potential mechanisms of virtual reality-mediated analgesia in postoperative and procedural pain among ICU patients nevertheless merit careful consideration.

With regard to mechanisms, converging preclinical and clinical evidence suggests that the analgesic effects of VR are mediated primarily through top–down cognitive and affective modulation. Experimental studies have shown that different immersive VR paradigms (exploratory/game-based versus mindfulness-oriented) significantly reduce experimental pain intensity and unpleasantness, with neural oscillatory changes indicating a reallocation of attentional resources within parietal–prefrontal networks and engagement of amygdala-centred emotion-regulation circuits (47). These central mechanisms may, in turn, influence autonomic and inflammatory pathways. In non-ICU populations, nature-based VR exposure has been associated with improved heart rate variability, consistent with reduced sympathetic arousal, while VR-facilitated activity has been linked to reductions in circulating IL-6 concentrations (48, 49). Although these data derive from non-critical care settings, they provide a plausible physiological framework through which VR might attenuate stress-related pain in ICU patients. Furthermore, VR interventions integrate cognitive-behavioral therapy, mindfulness and embodied techniques, potentially disrupting the pain-anxiety-sleep cycle through stress reduction, endogenous analgesia activation, and mood/sleep improvement (15).

ICU-focused trials are broadly consistent with this mechanistic profile: across recent systematic reviews, the most reproducible benefits of VR in critically ill patients relate to reductions in anxiety and improvements in sleep, whereas analgesic effects are less stable when pain is analysed as a secondary endpoint (50, 51). In cohorts with low baseline pain burden, whose predominant complaints are anxiety or general discomfort, VR may therefore confer chiefly psychological benefit (52). By contrast, in high-intensity contexts such as chest drain removal or wound care, our pooled analyses indicate a more robust analgesic signal, in line with meta-analytic evidence from general inpatient and outpatient populations showing that VR is most effective for acute procedural pain (17, 53).

Compared with general inpatient or outpatient populations, our findings reflect both the particularities of ICU cohorts and a degree of generalisability of VR’s analgesic effects. Large meta-analyses have shown that VR can reduce self-reported pain scores by approximately 0.8–1.5 points across a range of procedures (e.g., biopsy, endoscopy, dental interventions) and in postoperative pain settings (17, 54). Systematic reviews in chronic pain and musculoskeletal conditions likewise indicate that VR produces moderate reductions in pain alongside improvements in mood and physical function (26). By comparison, ICU patients typically have severe underlying organ dysfunction, complex pharmacotherapy and a highly stressful environment; analgesic strategies must balance comfort against the risks of delirium and haemodynamic instability (13). It is therefore unsurprising that the effect sizes observed in our review are more modest and the overall certainty of evidence lower, consistent with ICU-specific meta-analyses which report more robust effects of VR on anxiety and sleep than on pain itself (51). Notwithstanding the observed concordance with VR analgesia studies in general patient populations, the unique challenges of implementing VR-based pain management in critical care settings must be rigorously acknowledged.

Firstly, ICU patients experience highly complex and heterogeneous pain, driven by invasive procedures, inflammatory damage, underlying pathologies, and psychological stress (2). Pain expression fluctuates with disease severity and is influenced by sedation depth, delirium, and other factors, complicating the measurement and interpretation of intervention outcomes (13). Secondly, achieving rigorous blinding and standardized controls in study design is challenging, as VR experiences cannot be fully concealed, and studies often suffer from small sample sizes, inconsistent intervention timing, and content variations, which affect the reliability and replicability of conclusions (52). Thirdly, significant variability in ICU pain management practices exists, including differences in pain assessment tools (e.g., NRS, BPS, CPOT) and analgesia/sedation strategies, which serve as confounding variables. Fourthly, the instability of some ICU patients, including impaired consciousness, delirium, and hemodynamic instability, hinders sustained VR intervention or pain assessment, resulting in inadequate representation of high-risk populations in research (55). Moreover, current VR content lacks sufficient personalization for ICU-specific pain experiences (e.g., discomfort from mechanical ventilation or procedural pain), potentially limiting intervention efficacy (24). Finally, most studies focus on short-term outcomes within the hospital stay, lacking long-term assessments of chronic pain and overall efficacy (52). These challenges suggest that future research should broaden ICU population inclusion, standardize intervention protocols, incorporate objective pain metrics, optimize VR content, and extend follow-up periods to enhance methodological rigor and improve evidence quality.

Based on the existing literature and these findings, the research team recommend that future research focus on the following directions. Primarily, multicentre, rigorously blinded, well-designed RCTs are needed, with an emphasis on ICU scenarios involving severe pain, such as chest tube removal, wound care, early mobilization, positional adjustments, and large vessel puncture. Where possible, studies should consider expanding participant inclusion to include patients with mild to moderate sedation or awake mechanically ventilated patients to enhance external validity and ensure the representativeness of diverse patient groups. Concomitantly, a thorough assessment of the sources and types of pain in patients is required, alongside further standardization of VR interventions, including device types, content modules, treatment duration, and overall “dosage,” while exploring dose–response relationships and individualized prescription strategies. Future research could leverage artificial intelligence (AI) technologies to analyse patients’ emotional fluctuations and cognitive responses, dynamically adjusting VR content to maximize its analgesic effects (56). Tertiarily, outcome evaluations for VR interventions should extend beyond traditional subjective pain scores. A comprehensive collection of opioid and other analgesic usage, sedation scores, delirium, sleep quality, mental health outcomes, and adverse events is essential (57). Particularly in the context of chronic pain, AI algorithms should be used to analyse long-term follow-up data to assess the efficacy of VR interventions in long-term pain management and their impact on psychological well-being (58). Ultimatively, building upon standardized VR protocols and optimized conventional analgesia strategies, future research should adopt a more innovative and rigorous multidimensional pain assessment approach. Healthcare professionals should conduct a comprehensive analysis of different types of pain (e.g., acute pain from surgery, emotional regulation pain, and chronic pathological pain) to elucidate the physiological mechanisms behind how VR differentially modulates these types of pain. These initiatives will not only enhance the effectiveness of VR in ICU pain management but also provide more precise and personalized pain intervention strategies for clinical practice.

Despite signaling a potentially beneficial role of VR for ICU pain, this review has several unignorable limitations. The number of eligible studies was small and overall methodological quality was only moderate; VR protocols and pain measures were highly heterogeneous, and some QES lacked control groups or robust strategies to address confounding. Over half of the included studies (5/14) failed to report post-enrollment analgesic/sedative regimens, significantly limiting the interpretability of VR’s adjunctive effects against standardized pharmacological backgrounds. The lower certainty of evidence and potential publication bias may limit the applicability of these findings to a broader ICU population.

## Conclusion

This systematic review and meta-analysis suggests that virtual reality-assisted interventions using relaxation videos may help alleviate pain in ICU patients during chest drain removal and postoperative daily care. Given the quality of the evidence and potential publication bias, our findings should be interpreted as signal-generating evidence that VR is a promising adjunctive analgesic strategy in the ICU, rather than as definitive proof of efficacy; whether VR can meaningfully reduce ICU pain and analgesic requirements will require confirmation and refinement in higher quality studies. Future studies should evaluate VR in a broader patient population and clinical settings, employing standardized VR protocols and multidimensional assessments of pain and comfort. The use of analgesics and sedatives should be transparently reported, and multicentre trial designs are needed to clarify VR’s true role in ICU pain management.

## Data Availability

The original contributions presented in the study are included in the article/Supplementary material, further inquiries can be directed to the corresponding author.
