# Supplementary material for: The effects of virtual reality on pain relief in ICU patients: meta-analysis and systematic review
Source: Front Med (Lausanne). 2026 May 19;13:1792073. doi: 10.3389/fmed.2026.1792073 (PMC13226173; doi:10.3389/fmed.2026.1792073)
Supplement: Supplementary file 2 [file Supplementary_File_2.doc]

**S2-1: Cochrane's assessment items for RCT studies**

1. Method of randomization of cases.
2. Concealment of allocation.
3. Blinding of patients and investigators.
4. Blinding of outcome assessors.
5. Completeness of data.
6. Whether selective reporting.
7. Whether there is other bias

Refer to: <https://www.cochrane.org/.>

**S2-2: JBI Evidence Center’s quality evaluation items on QES studies**

1. Are the cause-and-effect relationships in the study clearly stated?
2. Are baselines comparable between groups?
3. Are baselines comparable between groups?
4. Is a control group established?
5. Are diversified measurements of outcome indicators conducted before and after the intervention?
6. Is the follow-up complete? If not, are the losses to follow-up reported and measures taken to deal with the problem?
7. Are the outcome indicators of each group of research subjects measured in the same way?
8. Is the measurement method of the outcome indicators reliable?
9. Are the data analysis methods appropriate?

Refer to: <https://jbi.global/critical-appraisal-tools.>
